# Paradoxical dopaminergic drug effects in extraversion: dose- and time-dependent effects of sulpiride on EEG theta activity

**DOI:** 10.3389/fnhum.2013.00117

**Published:** 2013-04-08

**Authors:** Mira-Lynn Chavanon, Jan Wacker, Gerhard Stemmler

**Affiliations:** Department of Psychology, Philipps-Universität MarburgMarburg, Germany

**Keywords:** electroencephalogram, theta activity, dopamine, sulpiride, agentic extraversion

## Abstract

Dopaminergic drugs frequently produce paradoxical effects depending on baseline performance levels, genotype, or personality traits. The present study for the first time aimed to specify the mechanisms underlying such opposite effects using the following recently reported scenario as an example: depending on the personality trait agentic extraversion (agentic facet, aE; i.e., assertiveness, dominance, ambition, positive emotionality) the selective dopamine D2 receptor antagonist sulpiride (200 mg) had opposite effects on resting posterior vs. anterior theta activity in the electroencephalogram (EEG). In order to better describe these opposite pharmaco-EEG effects and to generate hypotheses regarding the underlying mechanisms, we measured the EEG intermittently over 5 h in 80 healthy male volunteers extremely high or low in aE who had received either placebo or one of three doses of sulpiride (50, 200, or 400 mg). The findings suggest a model postulating stronger pre- vs. postsynaptic subreceptor effects in high aE individuals compared to low aE individuals. Future studies may now systematically apply the model to other examples of paradoxical dopaminergic drug effects and examine the molecular basis of individual differences in pre- vs. postsynaptic dopamine D2 subreceptor sensitivities and densities.

## Introduction

The effects of psychopharmacological manipulations of dopamine often show striking variability across individuals with the same drug (e.g., a dopamine agonist, a dopamine antagonist, caffeine) either increasing or decreasing measures of brain activity, cardiovascular activity, mood reaction and task performance depending on baseline values (Takeshita and Ogura, 1994; Bitsios et al., 2005), baseline performance (Mehta et al., 2004; Finke et al., 2010), dopamine synthesis capacity (Cools et al., 2009), working memory span (Kimberg et al., 1997, 2001; Mattay et al., 2000; Mehta et al., 2000; Gibbs and D'Esposito, 2005, 2006; Frank and O'Reilly, 2006; Wallace et al., 2011), dopaminergic genotypes (Mattay et al., 2003; Kirsch et al., 2006; Apud et al., 2007; Cohen et al., 2007; Roussos et al., 2009; Mueller et al., 2011; van Holstein et al., 2011; Rokem et al., 2012) and personality traits like psychoticism (Corr and Kumari, 2000), sensation seeking (Netter and Rammsayer, 1991; Hutchison et al., 1999), impulsivity (Corr and Kumari, 1997; Cools et al., 2007; Clatworthy et al., 2009; Zack and Poulos, 2009), and extraversion (Revelle et al., 1976; Rammsayer et al., 1993; Corr and Kumari, 1997; Rammsayer, 1998; Wacker et al., 2006; Wacker and Stemmler, 2006; White et al., 2006; Chavanon et al., 2007; Smillie and Gokcen, 2010). Understanding the precise mechanisms underlying such paradoxical effects would offer important insights into the dopaminergic foundations of various domains of personality. In the present study, we aim to explore these mechanisms using the strong moderating effect of extraversion on the consequences of the dopamine D2 receptor antagonist sulpiride on resting electroencephalogram (EEG) theta topography observed by Wacker et al. (2006) as an example.

The study by Wacker et al. (2006) aimed to test Wacker et al. (2006) suggestion that individual differences in a dopamine-based incentive motivational system, the Behavioral Facilitation System (BFS), underlies the personality trait of extraversion—more specifically its agentic facet (aE) encompassing drive, achievement striving, assertiveness as well as positive affective motivational states (elation, desire—wanting, energy) and vigorous and persistent goal-directed behavior in a wide range of achievement-related and social contexts^1^. Neurobiologically the BFS, which closely resembles Gray's (1994) Behavioral Approach System, is tied to the mesocorticolimbic dopamine system (MDS; Depue and Collins, 1999) which plays an important role in reward processing (Knutson and Cooper, 2005; Berridge, 2007) and projects from the dopaminergic cells of the ventral tegmental area (VTA) to limbic and cortical areas, such as the nucleus accumbens, cingulate cortex, prefrontal and orbitofrontal cortex (Depue and Collins, 1999; Wise, 2004; Bjorklund and Dunnett, 2007). Individual differences in functional properties of the MDS are thought to create respective differences in the BFS and hence in incentive motivation, approach/goal-directed behavior and aE (Depue and Collins, 1999). Consequently, aE should be associated with individual differences in brain dopamine.

Broadly supporting the aE-dopamine hypothesis, neuroimaging studies have reported associations between extraversion and activation at rest or in response to positive or rewarding stimuli within regions such as ventral striatum (i.e., caudate, putamen, nucleus accumbens), amygdala, medial orbitofrontal cortex (OFC), and anterior cingulate cortex (ACC; Canli et al., 2002; Kumari et al., 2004; Mobbs et al., 2005; Deckersbach et al., 2006) that Depue and Collins (1999) identified as particularly important in the dopaminergic circuitry of reward and approach behavior. In addition, psychopharmacological studies linked extraversion to individual differences in the hormonal response to a challenge with a selective dopamine receptor agonist (Depue et al., 1994; Depue, 1995) and molecular genetic studies have repeatedly found associations between extraversion and variants of dopaminergic genes (e.g., Reuter and Hennig, 2005; Reuter et al., 2006; Smillie et al., 2010).

Rather than focusing exclusively on genetic contributions and instead of using either expensive neuroimaging technology or invasive measurements of blood hormone levels Wacker et al. (2006) opted for an easily obtainable non-invasive EEG index, for which they expected both an association with aE and sensitivity to MDS activity: posterior vs. anterior EEG theta activity. In the meantime, the correlation between aE and this measure was replicated in several studies (Knyazev, 2009, 2010; Wacker and Gatt, 2010; Köhler et al., 2011). Recent studies using the low-resolution electromagnetic tomography algorithm (LORETA) suggest that major sources of posterior vs. anterior EEG theta index are likely located in the ACC (Knyazev, 2010; Chavanon et al., 2011) and the OFC (Knyazev et al., 2012). Also, initial molecular genetic studies have related posterior vs. anterior EEG theta activity to the COMT polymorphism (Val/Val carriers displayed increased posterior vs. anterior EEG theta activity and higher E scores; Wacker and Gatt, 2010) and the dopamine D2 receptor (DRD2) polymorphisms SNP19 rs1076560 and -141C Ins/Del (Köhler et al., 2011). For this index of resting posterior vs. anterior, EEG theta activity Wacker et al. (2006) observed that instead of the usual positive correlation with aE a significant negative correlation was present after administration of sulpiride (200 mg). Thus, sulpiride had completely opposite effects in individuals high vs. low in extraversion.

Besides aE, neuropharmacological studies have revealed an inverted U-shaped relation between working memory functioning and dopaminergic activity (see Arnsten, 1998, for a review). Given that both working memory and extraversion are currently thought to at least partly rely on brain dopamine, it seems reasonable to assume that dopamine connects the two in a systematic way. This suggestion is also corroborated by the fact that the MDS, vital to the concept of aE, is also the main dopaminergic projection to the frontal cortex and thus central to the inverted U-shaped relation between working memory and frontal dopamine. Recent studies revealed that extraversion predicts both working memory performance (Lieberman and Rosenthal, 2001; Chavanon et al., 2007) and working memory-related prefrontal brain activity (Gray and Braver, 2002; Kumari et al., 2004; Gray et al., 2005). Intriguingly, Wacker et al. (2006) reported that the disordinal effects on EEG theta topography were paralleled by paradoxical effects on 2- and 3-back working memory performance: whereas under placebo high aE showed shorter reaction times than low aE, which matched prior observations by Lieberman and Rosenthal (2001), sulpiride reversed these reaction time differences by speeding up low aE and slowing down high aE.

**Figure 1:**
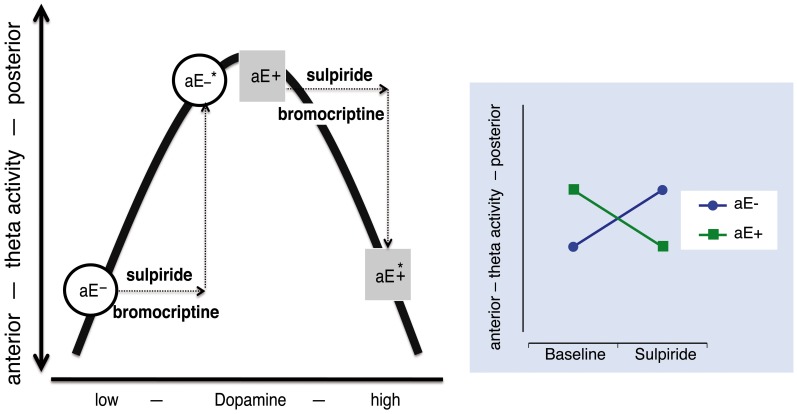
**The relationship between dopamine level and “resting posterior vs. anterior theta activity” follows an inverted U-shaped function.** High and low aE (aE+, aE−, respectively) differ in their initial position on this function. After an identical increase (arrows) of the dopamine level by either a dopaminergic agonist (e.g., bromocriptine) or a predominantly presynaptic dopaminergic antagonist (e.g., sulpiride) aE+ and aE− are shifted to positions (^*^) that mark opposing changes (disordinal interaction, see Substance × aE plot on the right side): aE− are shifted to medium and aE+ to high dopamine levels, resulting in more or less posterior vs. anterior theta activity, respectively. Such inverse U-shaped functions can be seen as the result of two underlying processes, but without such a specification it is merely a function of representation.

Such opposing or paradoxical effects of dopaminergic drugs have commonly been accounted for by the inverted U-shape principle (often *post-hoc*): Two groups (e.g., high vs. low aE) differ in their baseline levels of dopamine and hence occupy different initial locations on an inverted U-shaped function linking dopamine levels and the dependent variable. Administration of a dopaminergic drug (e.g., a D2 agonist) shifts the groups to different arms of the inverted U-shaped function, producing opposite drug effects for the two groups (Figure 1). However, more direct tests of the inverted U-shaped model that use varying drug doses are extremely rare. At the present time there are no empirical data available that elucidate the mechanisms on which such an inverted U-shaped curve between dopamine and posterior vs. anterior theta is based in the context of aE. Without specifying which distinct processes or mechanisms contribute to an inverted U-shaped relationship, it is just a function of representation.

**Figure 2:**
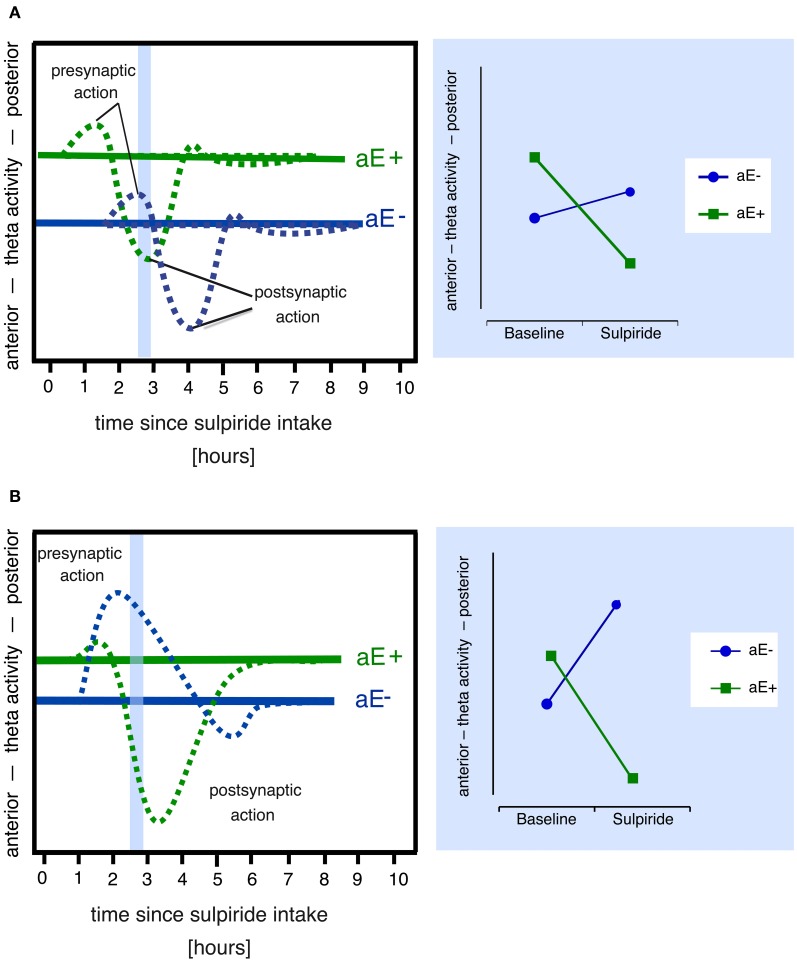
**Alternative models that might explain paradoxical sulpiride effects in aE based on the assumption of two time and dose-related processes (pre- and postsynaptic action).** Note that both models also assume that (1) high and low aE differ in their baseline levels of dopaminergic activity (i.e., pre- and postsynaptic receptor activity) and hence in their posterior vs. anterior theta activity scores and (2) posterior vs. anterior theta linearly tracks pre- vs. postsynaptic receptor activity. Panel **(A)** Due to different receptor sensitivities high and low aE (aE+, aE−, respectively) show time-shifted pharmacological actions: For example, at 2.5 h (area marked in light blue), the presynaptic action is still evident in aE−, but in aE+, the postsynaptic action already occurs. This results in a paradoxical effect (disordinal interaction, see resulting Substance × aE plot on the right side), since, compared to baseline, aE+ displays a shift toward anterior theta activity at 2.5 h (due to postsynaptic action), whereas aE− exhibits a shift toward posterior theta (due to presynaptic action). Panel **(B)** Differential receptor sensitivities could also produce stronger presynaptic than postsynaptic effects in aE− resulting in a net presynaptic effect and a shift toward posterior theta, whereas stronger sensitivity to postsynaptic action in aE+ results in a net postsynaptic effect and a shift toward anterior theta (marked light blue area), thus compared to baseline one observes a paradoxical effect (disordinal interaction, see resulting Substance × aE plot on the right side) at 2.5 h.

However, plausible alternative explanatory models for paradoxical effects can also be derived from the pharmacodynamic profiles of the dopaminergic drugs administered. For example, in the lower dosage range sulpiride enhances dopaminergic transmission and dopamine synthesis (Tagliamonte et al., 1975) as well as dopamine release by its antagonistic binding to the presynaptic D2/D3-autoreceptors (see review by Rankin et al., 2009), which explains its antidepressant impact, whereas at high doses postsynaptic blockade and reduced dopamine signaling predominate (Westerink and Devries, 1989; Serra et al., 1990; Kuroki et al., 1999). Hence, those two processes might contribute to the paradoxical effects observed by Wacker et al. (2006). A dose of 200 mg sulpiride—as used in the study by Wacker et al. (2006)—likely produces both pre- and postsynaptic effects, although presynaptic effects are thought to prevail (Mueller et al., 2011). Paradoxical dopaminergic effects in different individuals might arise from systematic differences in the time courses of pre- and postsynaptic drug effects. For example, due to different baseline levels of dopamine the responses to sulpiride might be shifted in time in high vs. low aE causing differing effects at a specific point of time (Figure 2A).

Finally, because sulpiride shows high affinity to D2 and D3 subreceptors (Strange, 2001), which are both highly expressed in midbrain structures and function at least partly as presynaptic autoreceptors (Rankin et al., 2009), and a lower affinity to D4 receptors, which are mostly expressed in prefrontal cortex, hippocampus, amygdala, and pituitary (Oak et al., 2000), sulpiride's pharmacological profile slightly expands with increasing doses as D4 receptors are additionally simulated^2^. Individual differences in any of these receptor densities or sensitivities might lead to paradoxical effects (Figure 2B), if they affect the balance of presynaptic (i.e., effects on DRD2 and DRD3 presynaptic autoreceptor subtypes) vs. postsynaptic (i.e., effects on DRD2, DRD3, and DRD4 postsynaptic receptor subtypes) drug effects, resulting in distinct patterns of response dominance.

Aiming to compare the models' (Figure 2) power to explain aE-driven paradoxical effects of sulpiride on posterior vs. anterior theta activity and 2-back working memory performance we measured the EEG intermittently over 5 h in individuals either extremely high or low in aE who had received either placebo or one of three doses of sulpiride (50, 200, 400 mg). We expected that responses of individuals high and low in aE would follow an inverted U-shaped function and/or that individuals high and low in aE differ systematically in the time course or dominance of sulpiride's pre- and postsynaptic effects.

## Materials and methods

### Participants

To select participants either extremely high or low in aE, we recruited a pool of *N* = 422 male, university or high school student volunteers, to fill in a German short scale of Tellegen's Multidimensional Personality Questionnaire designed to measure aE (see Wacker et al., 2006). In order to obtain greater homogeneity within aE groups the extreme group selection was based on the primary scales: participants scoring above the median in each of the three primary scales constituted the high aE extreme group, whereas participants with scores below or equal to the median in all three primary scales constituted the low aE extreme group. By virtue of this selection procedure the participants of the present study scored either above the top tercile (high aE) or below the bottom tercile (low aE) of the distribution of total aE scores. Preselected participants were further screened for their handedness (inclusion criteria: right-handed) and participants' health status was checked via interview: self-reports of chronic or acute diseases especially cardiovascular or gastrointestinal ailment or functional abnormalities of the liver or the kidney led to rejection from participation, as did habitual smoking of more than ten cigarettes per day, regular use of other drugs, and treatment with prescription drugs in the last 3 months. Furthermore, lifetime absence of psychiatric disorders was ascertained by a brief clinical interview based on DSM-IV criteria. *N* = 88 healthy male participants met the inclusion criteria and finally agreed to participate in the study. The study was approved by the Ethics Committee of the German Society for Psychology (Deutsche Gesellschaft fuer Psychologie) and performed in accordance with the Declaration of Helsinki. All volunteers gave written informed consent and were paid for participation (80€, approximately $120). Eight participants were excluded from statistical analysis, because they had less than 30 epochs of artifact-free EEG data due to excessive artifacts (eye and muscle movements; *n* = 5) or due to technical malfunction (*n* = 3) in more than two data recordings. Data are reported from 40 high-aE subjects (mean age = 22.70, *SD* = 2.53; range 19–30) and 40 low-aE subjects (mean age = 23.93, *SD* = 3.06; range 20–31). The participants of each aE extreme group were randomly assigned to either the placebo or one of the three D2 antagonist groups. A full description of the sample is given in Table 1.

**Table 1:** **Sample characteristics and descriptive statistics**.

	**Placebo**		**50**		**200**		**400**		**Significant effects**
	**high aE**	**low aE**	**high aE**	**low aE**	**high aE**	**low aE**	**high aE**	**low aE**	
Age	22.2 (0.8)	23.0 (0.7)	23.0 (1.0)	24.1 (1.0)	23.2 (0.9)	24.7 (0.8)	22.4 (0.6)	23.9 (1.3)	
Weight	76.6 (2.3)	71.4 (2.3)	79.1 (4.2)	80.4 (4.4)	76.0 (3.0)	83.3 (5.2)	78.9 (3.4)	80.3 (3.6)	
MAE	29.6 (2.1)	−7.4 (4.5)	27.6 (1.6)	−11.6 (3.4)	27.6 (2.6)	−6.7 (3.2)	27.3 (2.1)	−8.4 (4.4)	aE: 266^***^
MPQ NE	10.3 (2.8)	17.8 (2.2)	12.3 (2.7)	18.9 (3.7)	11.5 (2.5)	17.6 (2.7)	8.2 (1.4)	18.1 (2.5)	aE: 16.61^***^
EPQ-R E	19.8 (0.9)	10.8 (1.7)	20.7 (0.6)	11.7 (1.3)	19.4 (1.0)	12.1 (1.6)	19.2 (1.1)	12.1 (1.9)	aE: 75.63^***^
EPQ-R N	3.3 (0.7)	10.3 (2.0)	4.9 (1.4)	9.9 (1.9)	6.0 (1.0)	7.9 (1.2)	3.8 (1.0)	9.6 (1.8)	aE: 22.82^***^
EPQ-R P	8.0 (2.0)	10.5 (1.1)	8.7 (1.1)	11.9 (1.7)	6.7 (1.0)	8.9 (1.2)	8.6 (1.5)	7.7 (1.4)	
ZKPQ Act	8.7 (0.4)	6.9 (1.1)	10.8 (0.8)	6.0 (0.8)	11.4 (0.9)	7.2 (0.8)	10.5 (1.2)	5.1 (1.2)	aE: 37.85^***^
ZKPQ AH	6.1 (0.9)	5.6 (0.7)	5.9 (1.3)	5.5 (0.8)	6.8 (1.0)	6.6 (0.9)	4.1 (0.7)	5.7 (0.9)	
ZKPQ ImpSS	8.6 (1.4)	8.4 (1.7)	10.6 (0.9)	9.7 (1.5)	11.4 (1.0)	8.0 (1.2)	9.3 (1.5)	6.8 (0.8)	
ZKPQ NA	2.2 (0.9)	6.6 (1.6)	2.4 (0.9)	4.9 (1.4)	1.9 (0.6)	3.9 (0.8)	2.1 (0.6)	5.3 (1.3)	aE: 15.91^***^
ZKPQ Soc	11.2 (1.0)	6.0 (1.1)	10.0 (1.0)	7 (1.5)	11.0 (1.1)	6.3 (0.9)	10.4 (0.9)	8.0 (1.3)	aE: 22.65^***^
WMC	41.3 (4.1)	38.3 (5.1)	40.3 (6.1)	35 (6.0)	33.1 (5.1)	36.3 (5.6)	35.8 (4.5)	31.4 (7.0)	
CFT	26.3 (1.6)	28.3 (0.9)	31.6 (1.1)	27.3 (1.1)	26.3 (2.0)	28.5 (1.4)	29.0 (1.5)	25.9 (1.1)	aE × S: 3.06^*^
									E-P,E-200 < E50
									I-400, I-50 < E50

*Notes: Values given as means (standard errors). aE, Main effect agentic Extraversion, F_(1_, 72); aE × S: interaction of Trait aE and Substance, F_(3_, 72); EPQ-R, Eysenck Personality Questionnaire Revised (E, Extraversion; P, Psychoticism; N, Neuroticism); MAE, Marburg Agentic Extraversion Scale; MPQ NE, Multidimensional Personality Questionnaire–Negative Emotionality Scale; ZKPQ, Zuckerman–Kuhlman Personality Questionnaire (Act, activity; AH, aggression-hostility; ImpSS, impulsive sensation seeking; NA, neuroticism-anxiety; Soc, sociability); WMC, working memory capacity as measured by the automated span task; CFT, general fluid intelligence as measured by the short version of the Culture Fair Test (Scale 3)*.

* p ≤ 0.05,

*** *p ≤ 0.001, two-tailed*.

### Experimental design

The experimental design was a placebo-controlled, double-blind design defined by the between-subjects factors aE (high, low) and substance (placebo, D2 antagonist sulpiride dosages 50, 200, 400 mg) and the within-subjects factor time since substance (0.5, 1.5, 2.5, 3.5, and 4.5 h after substance intake).

All substances were delivered in capsules, which had the same appearance and were matched for weight to assure that the experimenter and the participant were blind to the pharmacological treatment. Sulpiride is a substituted benzamide derivative, shows high affinity within the nanomolar range to D2 and D3 receptors and a weaker affinity within the micromolar range to D4 receptors (Strange, 2001), and acts predominantly on the MDS (Mauri et al., 1996). Sulpiride appears to lack effects on norepinephrine, acetylcholine, serotonin, histamine, or gamma-aminobutyric acid receptors; it is rather slowly absorbed from the gastrointestinal tract, with peak serum levels occurring within 1–6 h after oral ingestion and elimination half-life is in the range of 3–10 h (Mauri et al., 1996). A major advantage of sulpiride is that adverse side effects are very rare (e.g., McClelland et al., 1990; Meyer-Lindenberg et al., 1997; Wacker et al., 2006). Regarding sulpiride's efficacy, the current literature suggests that low doses (50–150 mg) affect presynaptic autoreceptors producing its antidepressant efficacy, whereas higher doses (>800 mg) induce antipsychotic, postsynaptic D2 receptor effects (Serra et al., 1990). Based on this clinical profile it is assumed that 50 and 200 mg sulpiride could induce both pre- and postsynaptic D2 receptor effects but presynaptic effects predominate (see supportive data in Mueller et al., 2011). Based on the data of Mehta et al. (2008), 400 mg seems to induce stronger striatal occupancy compared to 200 mg sulpiride. In the same vein, a decreased striatal activation to reward seen with 400 mg sulpiride is in keeping with the hypothesis that inhibition of dopamine transmission (via postsynaptic effect) predominates 400 mg sulpiride (McCabe et al., 2011). In addition, the maximal prolactin response to 50 and 200 mg are time shifted (Sugnaux et al., 1983): the response to 50 mg sulpiride occurred 1 h later compared to 200 mg. Thus, the postsynaptic effects dominate later in time for low compared to high doses and consequently, presynaptic effects had to peak earlier in time for high compared to low doses.

### Two-back working memory tasks

In the present study, we employed the same 2-back paradigm as in Wacker et al. (2006). Participants are presented with a series of stimuli and asked to judge for each item as quickly and accurately as possible whether it matches the stimulus that preceded it by two places in the sequence (2-back task). Participants responded to each letter with their dominant right hand. For each of the five 2-back tasks a pseudorandomized sequence (30% target trials; 70% non-target trials) of 48 practice and 168 evaluated trials was generated. Of the non-target trials, 15% were lure trials, which are 1-back and 3-back repeats included as foils.

As stimuli we used single white letters (Times New Roman, 60 pt) each appearing in the center of a 15″-TFT display for 500 ms followed by a blank, black screen for another 1650 ms. Participants were expected to respond during this 2150 ms interval. The end of each trial was marked by a 350-ms auditory feedback, notifying whether the preceding reaction was correct and fast enough (“correct,” “incorrect,” “too slow”). “Too slow” referred to a correct reaction that was slower than a latency criterion, which was defined as the 90th percentile of the individual reaction time distribution for correct reactions during the practice trials of each 2-back task. For the computation of the individual percentiles, reaction times longer than three standard deviations above the individual mean were excluded. A new trial started right after the trial feedback (ITI = 0 ms; ISI = 2500 ms). For each of the five 2-back task presentations, the following performance measures were calculated: (a) the mean reaction time for correct reactions to targets, (b) the percentage of correct reactions to targets, and (c) the variability of reaction times for all correct reactions. For statistical analysis the reaction times and variability were square root transformed to normalize distributions. In order to control for unspecific attentional substance effects, we also introduced a 0-back task. The set-up was identical to the 2-back task, but participants were asked to indicate whether the present letter was a “q” or not. For lure trials we used 1-back and 2-back repeats as foils. It should be noted that Wacker et al. (2006) did not report significant effects for the 0-back task.

### Intelligence test, working memory capacity, and personality questionnaires

The participants completed the short version of Cattell's Culture Fair Test Scale 3 (CFT; Cattell and Weiß, 1971) in order to control for fluid intelligence as a possible confound and the automated version of the operation span task (Unsworth et al., 2005) in order to control for working memory capacity, which has already been shown to produce paradoxical effects with respect to the D2-receptor agonist bromocriptine (Kimberg et al., 1997). In addition, participants completed the German versions of the Zuckerman-Kuhlman Personality Questionnaire (ZKPQ; Zuckerman, 2002), the Eysenck Personality Questionnaire-Revised (EPQ-R; Ruch, 1999), and the MPQ-Negative Emotionality Scale (Tellegen and Waller, 2008). EPQ-R measures Eysenck's personality traits of E, Neuroticism, and Psychoticism. The ZKPQ measures Zuckerman's “Alternative Big Five,” Aggression-Hostility, Neuroticism-Anxiety, Sociability, Activity, and Impulsive Sensation Seeking.

### Setting and apparatus

The experiment was conducted in two adjacent rooms. The experimental room (4 × 3.4 m) had no windows, was air-conditioned (22°C), sound-attenuated, and had a largely non-technical appearance. Participants sat comfortably in a reclined position. A 15″-TFT monitor (Natcomp, Bad Homburg, Germany) and a response box (XQMS, Frankfurt, Germany) were placed in front of the participants. Electrodes were connected to a customized head box (Neuroscan, Sterling, VA), where EEG and electrooculogram (EOG) signals were preamplified with a gain of 30 (input impedance 10 MΩ). The adjacent room contained a 32-channel SynAmps 5083 amplifier (Neuroscan, Sterling, USA) and the technical equipment for experimental control and data acquisition. A Power Macintosh G4 performed data recording, data visualization, and data storage using LabView 5.0 software (National Instruments, Austin, USA). An IBM-compatible computer running Presentation 10.3 (Neurobehavioral Systems, Albany, USA) displayed stimuli and delivered prerecorded instructions.

### Procedure

The experiment was conducted in two separate sessions. In Session 1 the experimenter conducted a standardized clinical interview in order to check for lifetime absence of DSM-Axis I psychiatric disorders. Then participants completed the automated span task, the CFT and the personality questionnaires. Finally, they were trained on a attention control task and 2-back working memory to reduce potential practice effects for pharmaco-session (Wesnes and Pincock, 2002), in which EEG was recorded.

During Session 2 (starting at 8 a.m.; on average 1.5 days after session 1; range 1–9 days) the experimenter first conducted a semi-standardized interview to check protocol compliance (i.e., fasting, sleep duration and abstinence from alcohol, cigarettes, and caffeine for the last 12 h), and then positioned electrodes and explained emotion self-reports. The experimenter reminded the participants to sit quietly to help prevent artifacts in the EEG recordings and participants were told to relax with their eyes opened for a 10-min rest period with five embedded 1-min recordings. At the end of the rest period, participants filled in several self-reports on their current mood (Wacker et al., 2006). Participants then received either placebo or sulpiride together with a light breakfast. Thirty minutes after breakfast and substance intake, the experimental session started. It consisted of five blocks, with each of these blocks following the same set-up: first a 4-min rest period at which the EEG data reported here were recorded, then a 0-back attentional control task, next a 2-back working memory task to obtain behavioral measures for dopamine dependent cognitive processes, a 4 min post-task waiting phase terminated by a performance feedback, and finally a 30-min recreation period. A post-experimental semi-standardized interview concluded the experiment about 5.5 h after medication.

### Data acquisition, recording, and analysis

Vertical and horizontal electrooculogram (EOG) was recorded from four electrodes. EEG was recorded from 29 Ag/AgCl sintered ring electrodes (impedances <5 kOhm for EEG, <1 kOhm for the ground electrode AFZ; <10 kOhm for EOG) positioned in accordance with the International 10–20 system (Jasper, 1958) using an elastic electrode cap (Easy Caps, Germany). All sites were online referenced to Cz. EEG and EOG signals were amplified with a 32-channel SynAmps 5083 amplifier (EEG: gain 500; EOG: gain 100; input impedance 10 MOhm), digitally filtered (bandpass 1–50 Hz for EEG; lowpass 1 kHz for EOG; 50 Hz Notch filter) and stored (sampling rate: 2 kHz). Then signals were down-sampled to 250 Hz and converted to physical units. Subsequent pre-processing was carried out using BrainVision Analyzer 2 (Brain Products, Munich, Germany) and EEGLAB (Delorme and Makeig, 2004). Low-pass filters were located at 30 Hz and high-pass filters at 1 Hz. After visual rejection of data portions containing non-stereotyped artifacts (e.g., large muscle artifacts, swallowing, cable movement, etc.), concatenated EEG data were submitted to extended infomax-independent component analysis. Independent components reflecting eye blinks, lateral eye movements, line noise, and heartbeat pulses were identified visually and discarded by back-projecting all but these components to the data space. Corrupted channels flagged as artifact-contaminated for more than 1/4 of the recording were estimated using spherical spline interpolation (Perrin et al., 1989). In 2.13% of the data recordings Fz or Pz needed interpolation. Overall 2.25 % of the recorded channels were interpolated. Data portions and recordings with more than two corrupted channels were discarded. Next, all data epochs of 2.048 s were once again semi-automatically screened for artifacts.

All artifact free epochs were referenced to average electrodes and submitted to a Fast Fourier Transform (50% Hamming-windowed, padded symmetrically with zeros up to 1000 data points). The resulting estimates of power density (μV^2^/Hz; resolution 0.25 Hz) were clustered into theta (4.00–7.75 Hz) and delta (1.00–3.75 Hz) frequency bands both of which were shown to be sensitive to aE-related baseline/resting differences in posterior vs. anterior power distribution (Chavanon et al., 2011) and sulpiride (Wacker et al., 2006). Since the pattern of results for delta frequency data was almost identical to the theta pattern, we decided to restrict the presentation of results to the latter. As studies by Knyazev and colleagues (Knyazev, 2009, 2010; Knyazev et al., 2012) reported aE-related differences in posterior vs. anterior activity for higher frequency bands, we inspected broad alpha (8–12.75 Hz) and beta (13–29.75 Hz) frequency bands. All effects of interest (i.e., Substance, Substance × Time, Substance × Trait aE, or Substance × Trait aE × Time) were non-significant for both higher frequency bands, all *p*s > 0.5.

Power values were normalized by logarithmic transformation before statistical testing (see e.g., Davidson et al., 2000). The posterior vs. anterior EEG index was computed separately for each band as ln-transformed power at Pz minus ln-transformed power at Fz. In order to obtain reliable data recordings, only those with more than 30 artifact-free epochs (approximately 1 min) were kept (1.25% missing data). On average, EEG-analyses were based on 71.07 artifact-free epochs (*SD* = 19.44, range = 31–121) for post substance periods and 76.19 artifact-free epochs for the initial pre-drug baseline (*SD* = 22.18, range = 39–144). The number of artifact-free epochs was not associated with the Pz-Fz score, average correlations across data recordings *r*_(79)_ = −0.05. To control for individual baseline differences, the main statistical analysis was performed on reactivity scores computed by subtracting the pre-drug baseline. Prior to this subtraction, we ran a 2 × 4 ANOVA with Trait aE (2; high, low) and Substance (4; Placebo, 50, 200, 400 mg sulpiride) as group factors. Regarding posterior vs. anterior activity in theta band, there was neither significant effect of Substance, *F*_(3, 72)_ = 0.74, *p* = 0.53, nor an interaction effect of Substance × Trait aE, *F*_(3, 72)_ = 0.56, *p* = 0.65, for the initial, pre-drug resting period. However, as reported in detail in Chavanon et al. (2011), there were strong baseline differences between high and low aE in posterior vs. anterior theta activity, *F*_(1, 72)_ = 40.90, *p* < 0.0001, *d* = 1.51. High aE subjects showed more posteriorly located theta activity, whereas low aE depicted a more frontal pattern (Wacker et al., 2006, 2010). Posterior vs. anterior theta reactivity data was checked for normality prior to analysis using the Shapiro-Wilk test.

### Statistical data analysis

For all dependent variables a 2 × 4 × 5 repeated measures ANOVA with Trait aE (2; high, low) and Substance (4; Placebo, 50, 200, 400 mg sulpiride) as group factors and Time (5; 0.5, 1.5, 2.5, 3.5, 4.5 h since substance) as repeated factor was fitted in SAS/STAT (SAS Institute Inc., 1997) PROC MIXED. The error variance—covariance matrix was specified as completely general.

Significant ANOVA interactions were followed by a priori-specified contrasts tested with an α-level of 0.05, two-tailed. Contrasts for the posterior vs. anterior EEG theta reactivity scores were specified for three different interactions. These contrasts depict (a) the Substance × Time interaction regarding substance effects (placebo vs. sulpiride group) over time, (b) the Substance × Trait aE interaction focusing on (b1) substance effects (placebo vs. sulpiride group) within aE groups and (b2) dose-response relations within aE groups receiving sulpiride (a priori specified orthogonal contrasts for unequally spaced linear and quadratic dose-responses across 50, 200, and 400 mg), and finally (c) substance effects (placebo vs. sulpiride group) within aE groups over time (c1) to identify the first significant substance effect and (c2) to characterize time courses with orthogonal polynomial trends (linear, quadratic, cubic). Effect sizes (*r*_contrast_) for those latter repeated measures contrasts on temporal patterns (i.e., c2) were computed according to Furr and Rosenthal (2003); otherwise, Cohen's d was calculated.

## Results

### Pharmacological side-effects and blindness to substance groups

Participants did not report any adverse side effects. The ratings of nausea and dizziness averaged across experimental phases were very low (<1 on a 9-point scale with 0 = not at all applicable, 1 = not applicable) in all eight experimental groups. The percentage of participants, who guessed in a forced choice question in the post-experimental interview that they had received a pharmacologically active substance, did not differ between the substance groups [placebo: 40%, 400 mg sulpiride: 25%, 200 mg sulpiride: 30%, 50 mg sulpiride: 40%; *Chi*^2^ (3) = 1.51, *p* = 0.68]. When asked to evaluate the confidence in their guess, none of the participants reported to be 100% sure (*M* = 66%, *SD* = 19%). Thus, it can be concluded that the participants were blind to the experimental condition as intended.

### Changes in posterior vs. anterior activity

A significant main effect for Trait aE indicated that low compared to high aE showed a shift toward more posterior vs. anterior EEG theta [*F*_(1, 72)_ = 52.85, *p* < 0.0001, *M* = 0.076 and *M* = −0.069, *SEM* = 0.014 for low and high aE]. Furthermore, we observed a significant main effect of Time [*F*_(4, 72)_ = 9.95, *p* < 0.0001], which was best described by a linear trend toward more anterior vs. posterior theta across time [*t*_(72)_ = −5.65, *p* < 0.001; quadratic and cubic trends were non-significant, *p*s > 0.08]. The significant interaction effect of Trait aE × Time [*F*_(12, 72)_ = 6.73, *p* < 0.0001] could be traced back to diametrically opposed quadratic trends [*t*_(72)_ = −5.58, *p* < 0.0001]. However, these effects were further qualified by significant higher order interaction effects (see below).

A Substance main effect [*F*_(3, 72)_ = 19.23, *p* < 0.0001] revealed a linear dose response with 50 mg sulpiride inducing a shift toward stronger posterior theta and 400 mg inducing a shift toward a stronger anterior theta [*t*_(72)_ = 3.89, *p* < 0.0025, quadratic ns]. In addition, the expected interaction of Substance × Time was observed [*F*_(12, 72)_ = 6.73, *p* < 0.0001]. This interaction effect was due to stronger quadratic trends over time for sulpiride groups compared to placebo [*t*_(72)_ ≥ |1.78|, *p* ≤ 0.01], with an opposite direction for 50 mg sulpiride compared to 200 [*t*_(72)_ = 4.06, *p* = 0.0001] and 400 mg, [*t*_(72)_ = 4.48, *p* < 0.0001].

Most importantly, the predicted interactions of Substance × Trait aE [*F*_(3, 72)_ = 18.81, *p* < 0.0001] and Substance × Trait aE × Time [*F*_(12, 72)_ = 3.74, *p* < 0.001], were also highly significant. These expected aE based modulations of sulpiride effects were subsequently probed by a priori contrasts.

#### A priori contrasts

**Figure 3:**
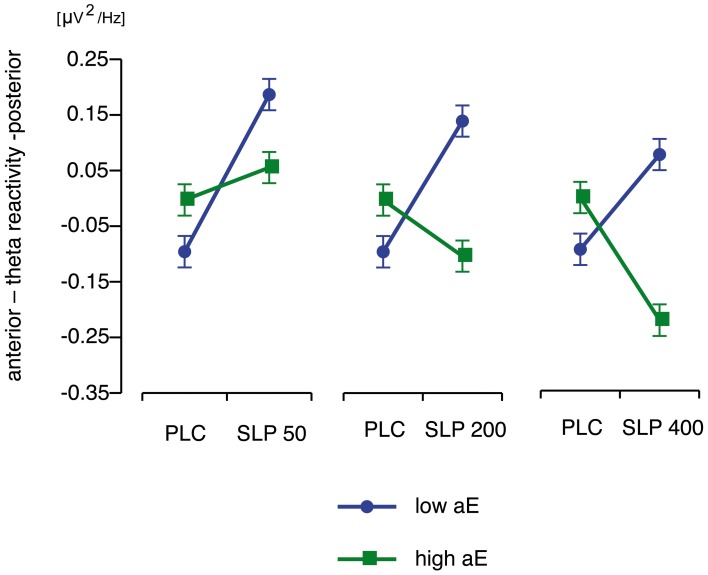
**Disordinal interactions of aE × Substance on posterior vs. anterior theta reactivity (*M* and *SEM* values) contrasting high and low aE groups which received placebo (PLC) with each of the three different doses of sulpiride (SLP; 50, 200, or 400 mg)**.

***Substance × Trait aE***. The tests of the central a priori contrasts are documented in Table 2. The corresponding means (and *SEM*s) are shown in Figure 3. High aE participants, who had received 200 and 400 mg sulpiride, exhibited a significant shift toward more anterior vs. posterior theta distribution compared to placebo (*d* = 0.60 for 200 mg and *d* = 1.30 for 400 mg) and the opposite was true for low aE (*d* = −1.39 for 200 mg and *d* = −1.01 for 400 mg). Compared to placebo the lowest dose of 50 mg sulpiride resulted in changes toward more posterior theta that were highly significant in low aE (*d* = −1.67), but non-significant in high aE (*d* = −0.35). Dose-response analyses using orthogonal polynomials for unequally spaced factor levels revealed that linear dose-responses were stronger for high aE than for low aE [*t*_(72)_ = 2.89, *p* = 0.005, *d* = 0.68]. For quadratic trends all contrasts were non-significant (*p*s > 0.25).

**Table 2:** **Substance effects within and between high and low aE in posterior vs. anterior theta reactivity: *t*-values of contrasts (effect size *d*)**.

	**aE contrasts**		
**Sulpiride effect**	**High aE**	**Low aE**	**High vs. low**
Placebo-50	−1.46 (−0.34)	−7.08^***^ (−1.67)	3.98^***^ (0.94)
Placebo-200	2.54^*^ (0.60)	−5.90^***^ (−1.39)	6.92^***^ (1.63)
Placebo-400	5.51^***^ (1.30)	−4.28^***^ (−1.01)	5.97^***^ (1.41)

Notes: aE, agentic Extraversion; 50, 200, and 400 refer to the groups that received 50 mg, 200 mg, or 400 mg sulpiride. *N* = 80. *df* = 72,

* p = 0.05,

*** *p ≤ 0.001, two-tailed*.

**Figure 4:**
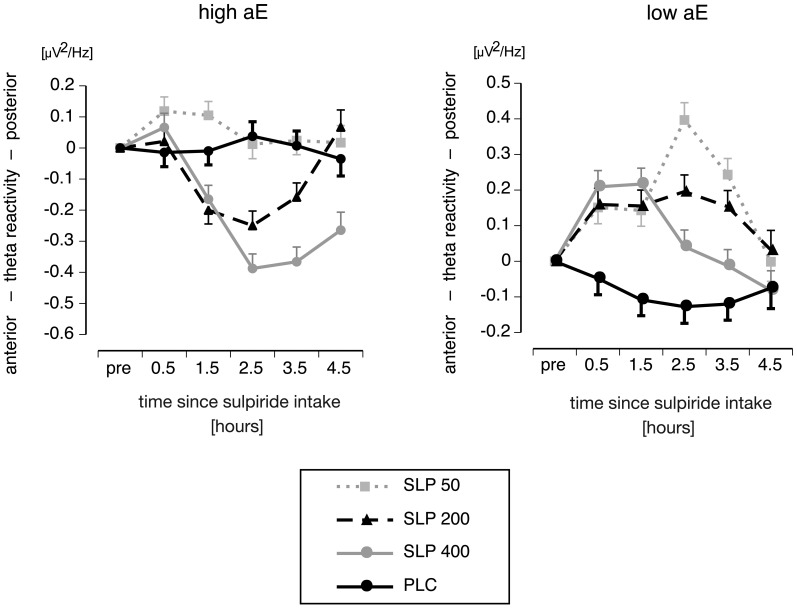
**Interaction of aE, Time and Substance on posterior vs. anterior theta reactivity (*M* and *SEM* values) focusing on time courses of posterior vs. anterior theta reactivity for high (upper panel) and low aE groups (lower panel), who received either placebo (PLC) or one of three different doses of sulpiride (SLP; 50, 200, or 400 mg)**.

***Substance × Trait aE × Time***. As expected neither placebo group (high or low aE) showed any significant trends across time (all *t*(72) values ≤ |0.95|, *p*s ≥ 0.35). Figure 4 displays the differential time courses of reactivity scores observed for high and low aE within placebo and sulpiride groups. The associated a priori contrasts are provided in Table 3. All aE groups that received sulpiride exhibited significant substance effects. All those effects—except for high aE 50 mg sulpiride—remained significant for at least three consecutive recording times and, thus, lasted for at least 2 h (see Table 3).

**Table 3:** **Time course of substance effects within and between high and low aE for posterior vs. anterior theta reactivity: *t*-values of contrasts (effect sizes)**.

				**Time since substance [h]^a^**					**Trends across time^b^**		
**Substance × aE Contrast**		**0.5**	**1.5**	**2.5**	**3.5**	**4.5**	**Linear**	**Quadratic**	**Cubic**
Placebo-50	High aE	−2.06^*^ (−0.48)	−1.82 (−0.43)	0.39 (0.09)	−0.25 (−0.06)	−0.67 (−0.16)	1.15 (0.14)	−1.14 (0.14)	−0.67 (0.08)
	Low aE	−3.08^**^ (−0.73)	−3.98^***^ (−0.94)	−7.78^***^ (−1.83)	−5.68^***^ (−1.34)	−0.92 (−0.22)	0.574 (0.07)	3.82^***^ (0.42)	2.11^*^ (0.25)
	High vs. low	0.73 (0.17)	1.53 (0.36)	5.83^***^ (1.37)	3.79^***^ (0.89)	0.19 (0.05)	0.40 (0.05)	−3.82^***^ (0.40)	−1.98 (0.23)
Placebo-200	High aE	−0.55 (−0.13)	3.01^**^ (0.71)	4.35^***^ (1.03)	2.52^*^ (0.59)	−1.31 (−0.31)	−0.76 (0.09)	−4.61^***^ (0.49)	−0.11 (0.01)
	Low aE	−3.22^*^ (−0.76)	−4.18^**^ (−0.98)	−4.90^***^ (−1.15)	−4.27^***^ (−1.01)	−1.34 (−0.31)	0.83 (0.10)	2.06^*^ (0.24)	0.83 (0.10)
	High vs. low	1.88 (0.44)	5.08^***^ (1.20)	6.54^***^ (1.54)	4.79^***^ (1.13)	0.04 (0.01)	−1.12 (0.14)	−4.72^***^ (0.50)	−0.67 (0.08)
Placebo-400	High aE	−1.24 (−0.29)	2.46^*^ (0.58)	6.44^***^ (1.52)	5.58^***^ (1.31)	2.87^**^ (0.68)	3.64^***^ (0.41)	−3.56^***^ (0.40)	−0.58 (0.07)
	Low aE	−3.98^***^ (−0.94)	−5.15^***^ (−1.21)	−2.55^*^ (−0.60)	−1.68 (−0.40)	0.08 (0.02)	3.35^**^ (0.38)	0.98 (0.12)	−0.82 (0.10)
	High vs. low	1.94 (0.46)	5.38^***^ (1.27)	6.36^***^ (1.50)	5.19^***^ (1.22)	1.97 (0.46)	0.40 (0.05)	−3.26^**^ (0.37)	0.14 (0.02)

*Notes. N = 80. aE, agentic Extraversion. 50, 200, and 400 refer to the groups that received 50 mg, 200 mg, and 400 mg sulpiride. df = 72 for simple Substance effects in aE (i.e., effects of each sulpiride dose vs. placebo for high and low aE) and Substance × aE effects (tetrad differences; i.e., difference of the effects of each sulpiride dose vs. placebo in high vs. low aE) at each time point, df = 67 for trends across time*.

a *Effect size Cohen's d for simple Substance and Substance × aE effects at each time point (between subjects)*.

b *Effect size r_contrast_ for trends across time (linear, quadratic, and cubic pattern; repeated measures)*.

* *p ≤ 0.05*,

** *p ≤ 0.01*,

*** *p ≤ 0.001, two-tailed*.

Under 50 mg, high aE significantly differed from their placebo control group as early as 0.5 h after intake. Notably, for all high aE groups the first response to sulpiride was a shift toward posterior theta activity, although this shift was not significant for 200 and 400 mg sulpiride. Contrasts for high aE participants further revealed that compared to placebo a first statistically reliable response to 200 and 400 mg sulpiride occurred after 1.5 h. While for 200 mg the substance-induced shift toward anterior theta lasted for about 2 h (1.5–3.5 h after intake), it lasted 3 h for 400 mg (1.5–4.5 h after intake).

Substance effects occurred earlier in time for low than high aE: half an hour after substance intake there was a reliable shift toward posterior theta in all sulpiride groups. While for 400 mg this effect lasted approximately 2 h, substance effects of 200 mg and 50 mg were significant for 3 h.

Maximal posteriorization response to 50 and 200 mg sulpiride was delayed compared to 400 mg in low aE (2.5 vs. 1.5 h for 50/200 and 400 mg, respectively). It should be noted that the linear dose-response pattern for low aE changed: whereas from 0.5 to 1.5 h after intake 400 mg induced stronger effects than 50 mg, this was reversed at 2.5 and 3.5 h. For high aE maximal anteriorization responses to 400 mg and 200 mg occurred 2.5 h after intake. The linear dose-response pattern (400 mg > 200 mg > 50 mg) remained stable from 2.5 h on.

Characterizing time courses by polynomial trends revealed that high and low aE depicted opposing quadratic time components, and this was true for all sulpiride groups (see *t*-values in Table 3). High aE showed an increase in *anterior* vs. posterior theta followed by a decrease, whereas low aE exhibited an increase in *posterior* vs. anterior theta followed by a decrease. Within the 50 mg sulpiride groups, low aE additionally showed a cubic component that was mainly due to a sharp rise to posterior theta at 2.5 h and a significant reduction in posterior theta at 4.5 h (see Figure 4), whereas nonlinear trends for high aE were not significant. After 400 mg, both low and high aE groups depicted an additional linear trend over time.

### Working memory performance: 2-back task

Neither reaction time for correct target responses nor percentage of correct target responses in the five 2-back tasks showed any effect related to Substance or aE. In contrast to the percentage of correct target responses, for which no effects were observed, reaction times speeded up with each hourly task block [*F*_(4, 72)_ = 21.25, *p* ≤ 0.0001].

For reaction time variability we observed an main effect of Time [*F*_(4, 72)_ = 4.61, *p* ≤ 0.003], which was described by a cubic trend across time [*t*_(72)_ = 3.28, *p* < 0.01, all other trends *p*s > 0.07]. Furthermore, a significant effect of Substance [*F*_(3, 72)_ = 3.28, *p* = 0.026] was revealed. Variability was lower under placebo compared to all doses of sulpiride [*t*_(72)_ values > 2.23, *ps < 0.03*], while there were no significant differences between the three sulpiride dosages [*t*_(72)_ values ≤ |0.49|, *p*s ≥ 0.62; average variability in ms (SD): placebo 80 (26), 50 mg sulpiride 103 (34), 200 mg sulpiride 97 (30), 400 mg sulpiride 101 (36)].

Controlling for attentional effects in the three performance measures as measured with the 0-back task (entered as repeated measures covariate, see Winer, 1971) did not change the pattern of results.

Grand means for the performance measures are given in Table 4 for each hour. Note that even in the first task block despite a comparable percentage of correct target responses, both reaction time and reaction time variability were considerably lower than in the previous study by Wacker et al. (2006), possibly due to the practice session on a separate day introduced in the present work [reaction time (*SD*): 444 ms (100) vs. 600 ms (149), reaction time variability (SD): 98 (31) ms vs. 157 ms (53), correct target responses (SD): 74% (13.3) vs. 72% (17) for present vs. Wacker et al., 2006, respectively].

**Table 4:** **Grand means (SD) of the performance measures in 2-back task for each hour since substance intake**.

**Hours since substance intake**	**Target reaction time (ms)**		**Reaction time variability (ms)**		**Correct target reactions (%)**	
1	444	(100)	98	32	74	(13.3)
2	423	(98)	95	35	73	(14.4)
3	409	(92)	94	33	74	(14.8)
4	410	(102)	100	37	72	(15.5)
5	397	(97)	94	31	74	(14.4)

### Specificity to aE

To check whether the effects of sulpiride on posterior vs. anterior EEG theta activity were modulated by other (correlated) personality traits (either EPQ-R-neuroticism, ZKPQ-aggression/hostility, ZKPQ-impulsive sensation seeking, MPQ-negative emotionality), age, weight, general fluid intelligence or working memory capacity, we calculated a series of ANCOVAs using the statistical model described above, but now entering in turn each variable as an additional covariate, its two-way interaction with Substance, its two-way interaction with Time and its three-way interaction with Substance and Time. The results of these supplementary analyses revealed that the interactions Substance × Trait aE and Substance × Trait aE × Time remained significant [Trait aE × Substance: *F*_(3, 68)_ ≥ 10.78, *p* ≤ 0.0001; Trait aE × Substance × Time: *F*_(12, 68)_ ≥ 2.31, *p* ≤ 0.015], indicating that the effects reported above are indeed specific to aE.

## Discussion

The present study focused on paradoxical dopaminergic effects and confirmed that the effect of sulpiride on posterior vs. anterior theta activity strongly depends on aE. Low aE showed more frontally distributed theta than high aE, and under 200 and 400 mg sulpiride this difference was reversed: high aE showed a shift toward anterior theta, but low aE, a shift toward posterior theta. Furthermore, we found marked aE-related response differences across time. Thus, the present findings support the basic idea that besides general responses to pharmacological agents and static models like an inverted U-function, time aspects of pharmacological effects contain valuable information regarding the biological basis of Extraversion. While EEG theta activity proved sensitive to the paradoxical effects of sulpiride on high and low aE, such effects could not be detected for 2-back working memory performance. Based on the present findings we will discuss in detail differential pre- and postsynaptic responses in high and low E as one possible explanatory mechanism after briefly refreshing the most important features of sulpiride's pharmacodynamic profile.

### The pharmacodynamics of sulpiride doses

Sulpiride shows high affinity within the nanomolar range to D2 and D3 receptors and a weaker affinity within the micromolar range to D4 receptors (Strange, 2001), and acts predominantly on the MDS (Mauri et al., 1996). Regarding sulpiride's biphasic action and clinical efficacy, the literature suggests that low doses (50–150 mg) affect presynaptic D2/D3-autoreceptors (see review by Rankin et al., 2009) producing its antidepressant efficacy, whereas higher doses (>800 mg) induce antipsychotic, postsynaptic D2 receptor effects (Westerink and Devries, 1989; Serra et al., 1990; Kuroki et al., 1999). Based on this clinical profile it is assumed that 50 and 200 mg sulpiride used here could induce both pre- and postsynaptic D2 receptor effects but presynaptic effects predominate (Mueller et al., 2011). Furthermore, a dose of 400 mg induced stronger striatal occupancy compared to 200 mg (Mehta et al., 2008) and produced a marked decrease in striatal activation to reward (McCabe et al., 2011). These data suggest that the inhibition of dopamine transmission (via postsynaptic effects) predominates the effects of 400 mg sulpiride. In a nutshell, there is a dose-dependent biphasic action that relates to the balance of pre- to postsynaptic effects (50 mg > 200 mg presynaptic vs. 400 mg postsynaptic predominance). In addition, pharmacokinetic data showed that within the tuberoinfundibular system the maximal prolactin response to 50 and 200 mg are time shifted (Sugnaux et al., 1983): the response to 50 mg sulpiride occurrs1 h later compared to 200 mg. Thus, postsynaptic effects rush in or dominate later in time for low compared to high doses. Hence one could expect that in the present study 400 mg reach the plasma levels for pre- and postsynaptic effects in the MDS earlier in time compared to 200 mg and the postsynaptic effects to 50 mg -if at all- will be observed last.

### Evaluation of inverted U-shaped model (Figure 1)

**Figure 5:**
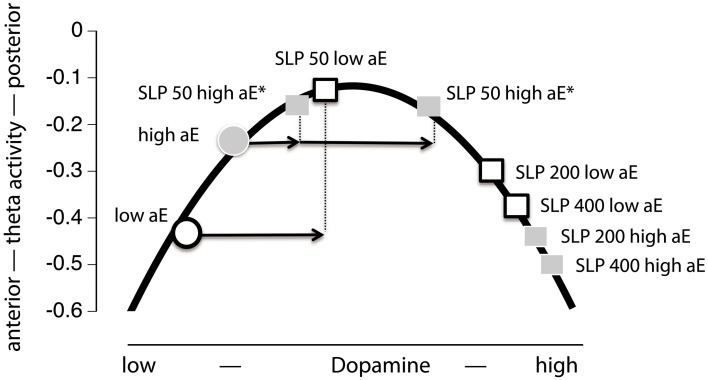
**Projection of the present Substance × aE interaction effect onto an inverted U-shaped relationship between posterior vs. anterior theta activity and dopamine level (Figure 1).** Postulating such an inverted U-shaped function implies that sulpiride (SLP) acts presynaptically. In order to facilitate interpretation we used mean values based on posterior vs. anterior theta activity across 0.5–3.5 h (i.e., time range of maximal substance effects). Thus, by increasing dopamine level through presynaptic sulpiride action, the typical posterior vs. anterior theta activity values of high and low aE observed under placebo (circles) are shifted to different locations on this function (squares). For high aE under 50 mg sulpiride, the position on the inverted U-shaped function could either be on the left or the right arm (indicated by asterisks). The arrows indicate aE-based differences in the magnitude of presynaptic action (*x*-axis) to 50 mg, which must be assumed—in either case—in order to accommodate the observed Substance × aE interaction pattern within the inverted U-shape model.

This model assumes that dopamine and posterior vs. anterior theta are linked by an inverted U-shaped function and that equal doses of sulpiride influence dopamine levels in the same, commensurate direction for high and low aE. This implies that all observed effects are necessarily presynaptic, increasing dopamine levels. Thus, the model would make the following prediction for the present data: low aE are typically located on the low dopamine left side of the inverted U and sulpiride shifts them up the ascending limb through presynaptic blockade. The same mechanism pushes high aE up to the top of the curve and beyond (descending limb). When focusing the time points where substance effects were most pronounced (0.5–3.5 h; see Figure 5), this predicition fits the empirical data, although the position of high aE for the 50 mg dose is ambiguous and the size of shifts differ between high and low aE for the 50 mg dose. In addition, this model focuses on the interaction effect of Trait aE and Substance, and hence, it cannot explain any of the effects qualified by time.

### Evaluation of alternative explanatory models (Figure 2)

Model A assumes that sulpiride produces pre- (50 mg) or pre- and postsynaptic (200, 400 mg) responses of similar magnitude in low and high aE groups that are, however, shifted in time. Applied to the present data model A would suggest that more posterior vs. anterior theta directly tracks dopamine levels (posterior shift = presynaptic blockade, dopamine increase; anterior shift = postsynaptic blockade, dopamine decrease). In high aE the presynaptic effects are visible under 50 mg at 0.5 h. For higher doses significant presynaptic effects should have appeared before 0.5 h. Postsynaptic effects for both higher doses started as early as 1.5 h. Conversely, in low aE only presynaptic effects were evident and enduring. In neither group postsynaptic net effects were found within 4.5 h. Although model A can principally account for the findings, it is necessary to assume that significant portions of the responses occurred within the first 30 min for high aE (missing parietalization) and after 4.5 h for low aE (missing anteriorization). These time points were not included in data sampling here. Given that serum levels of sulpiride have been reported to peak within a widely varying interval (1–6 h), a direct test of these assumptions in future studies seems warranted in order not to dismiss the model prematurely. However, the observed differences in response magnitude for high and low aE are not covered by the model exclusively assuming a aE-related time shift in responses.

Model B holds that in high aE postsynaptic effects should dominate at least for the two higher doses of sulpiride, whereas for low aE net presynaptic effects should be observable for all doses, but particularly for the 50 mg dose. In high aE 50 mg should generate at least a small presynaptic response. Once again assuming that posterior vs. anterior theta directly tracks dopamine levels the observed pattern closely matches these predictions: low aE only showed presynaptic effects peaking earlier for the higher dose than for the lower ones, whereas high aE primarily showed postsynaptic effects for the two higher doses, with 400 mg peaking later than the presynaptic effects observed for low aE. In high aE we obviously observed an initial presynaptic net effect after 0.5 h for 50 mg, but presynaptic effects for 200 and 400 mg were non-significant. An explanation for this pattern in high aE might be that even under 50 mg presynaptic effects are opposed (but never outweighed) by postsynaptic effects in the time interval around 1–4.5 h during which peak sulpiride plasma levels most likely occur. Alternatively, the lack of evidence for more enduring net presynaptic effects in high aE under 50 mg may be due to ceiling effects in our EEG measure (i.e., high aE may have already demonstrated a maximally posterior distribution of theta activity under placebo).

It should be noted that aE-related differences in the D2-like subreceptors DRD2, DRD3, and DRD4 might account for this pattern. For example, a simple aE-related difference in postsynaptic D4 receptors might lead to aE × Substance × Time interaction because sulpiride's pharmacological profile expands across time and additionally stimulates D4 receptors: if low aE have less DRD4 receptors than high aE they would -in contrast to high aE- show less DRD4 related postsynaptic effects that typically rush in when sulpiride reaches micromolar concentrations. Taken together, model A can accommodate some of the observations whereas model B can explain the complete pattern of findings although the precise contribution of D2-like receptors is not elucidated with the present research.

### Integration of recent research

Investigating the posterior vs. anterior EEG theta activity with polymorphisms related to dopamine D2 receptor functioning, a recent study showed that SNP19 rs1076560, which is implicated in the regulation of two isoforms of the DRD2 receptor (Zhang et al., 2007), and -141C Ins/Del rs1799732, which has been associated with altered expression of the DRD2 in the striatum, were significantly associated with posterior vs. anterior EEG delta/theta activity (Köhler et al., 2011). Particularly, the SNP19 rs1076560 polymorphism might be relevant to the present data and their interpretation, because this polymorphism is associated with relative expression of the DRD2 long isoform (D2L), which is mainly postsynaptic and the DRD2 short isoform (D2S), which is mainly presynaptic and serves as an autoreceptor regulating dopamine synthesis and release (Usiello et al., 2000) in the frontal cortex. Furthermore, D2S receptors are the most abundant autoreceptor subtype in the midbrain (Khan et al., 1998) and provide potent inhibition of dopamine release. However, the SNP19 rs1076560 T allele shifts splicing from short to long receptors, decreasing the D2S/D2L ratio relative to the G allele and therefore the T allele is associated with putatively greater levels of midbrain dopamine. Köhler et al. (2011) reported that the T allele compared to the G allele was associated with less posterior vs. anterior EEG delta/theta activity and carriers had numerically lower scores in Extraversion. Combining those findings and the hypothetic principles of tonic and phasic dopaminergic activity (Grace, 1991) would lead to the following prediction: low aE might more frequently be carriers of the T-allele and consequently have higher tonic midbrain dopamine levels. High dopamine levels result in a lower (phasic) responsivity of postsynaptic receptors, leading sulpiride's presynaptic effects to prevail. For high aE the lower dopamine level results in higher responsivity of postsynaptic receptors, leading to sulpiride's postsynaptic effects to prevail. This is exactly what we found in the present study. Combining the present pharmacological design with the genetic approach used in Köhler et al. (2011) could provide a direct test of this model.

Regarding the functional significance of the posterior vs. anterior theta measure, there are some aspects we would like to point out. As anterior theta is generated in ACC (Ishii et al., 1999), we recently probed the ACC as a potential source of posterior vs. anterior theta and found that especially theta in the rostral portion (rACC) was strongly associated with low values in our EEG measure (Chavanon et al., 2011). In line with the present results, ACC is known to respond to dopaminergic challenges (Vollm et al., 2004). Interestingly, high levels of inhibitory rACC delta/theta activity (i.e., presumably low ACC activity) have been associated with both blunted nucleus accumbens reward responses and anhedonia, i.e., reward-insensitive behavior and blunted positive emotionality or, arguably, extremely low aE (Wacker et al., 2009). Furthermore, ACC activity predicts the psychopharmacological treatment response in depressive patients (Korb et al., 2009). Thus, low aE individuals may have demonstrated a sulpiride-induced “antidepressive” reaction in rACC mirrored by posterior vs. anterior theta. Pizzagalli (2011) recently argued that the rACC plays a key role in treatment outcomes due to its prominent position in the default network. He related the antidepressive rACC response to adaptive self-referential processing which parallels our suggestion that posterior vs. anterior theta (and low inhibitory rACC theta) is positively associated with optimistic future-oriented mentation about one's self and personally significant issues (Chavanon et al., 2011). However, data by Knyazev and colleagues (Knyazev, 2012, 2013; Knyazev et al., 2012) and unpublished data from our group supported the idea that aE is associated with higher theta activity in the default mode network.

Regarding the posterior component of the posterior vs. anterior theta index, Chavanon et al. (2011) reported that inferior parietal and insular cortex were negatively associated with aE. Those results converge with a recent study showing that the insula is inversely related to the willingness to work for reward (Treadway et al., 2012), which is a major facet of aE (i.e., persistent reward striving). Because the insula receives dopaminergic innervation (Gaspar et al., 1989) and expresses D1-like and as well as to a lesser extent D2-like receptors (Hurd et al., 2001), it can be speculated that—in addition to the rACC—the insula might have contributed to the results presented here. Other structures which might have contributed could be the inferior parietal cortex, precuneus and posterior cingulate which were a) functionally connected to the striatum under resting conditions (Di Martino et al., 2008) and b) recently linked to extraversion (Knyazev, 2013). However, it should be kept in mind that based on its neuroanatomy, the dopaminergic system exerts its influence more strongly on frontal brain structures than on posterior brain structures (Cools and D'Esposito, 2011). Thus, compared to Chavanon et al. (2011), the present data presumably rely more heavily on the anterior component of the theta index due to the pharmacological manipulation of the MDS.

### Limitations

The following caveats should be noted: (1) The present study was conducted with male participants and thus leaves generalizability to women open. (2) The assessment of sulpiride effects was limited to posterior vs. anterior theta activity and 2-back working memory performance. In contrast to Wacker et al. (2006) who reported diametrically opposite effects on multiple levels, the present paradoxical effects were restricted to the EEG measure. Unfortunately, we cannot explain the lack of effects on working memory performance. Because other biological indicators such as plasma dopamine levels were not assessed, a validation of the EEG measure with other dopamine biomarkers or a dopmamine-related cognitive phenotype is missing here. (3) Although sulpiride is a highly selective dopamine D2 receptor antagonist, we cannot rule out that the effects observed are due to interactions with other neurotransmitter systems rather than purely dopaminergic. (4) Furthermore, we cannot rule out that there were substance effects before our initial measurement at 30 min and after the final measurement at 4.5 h. Thus, a definitive conclusion concerning the time-course model requires a study with an even more extended recording interval. (5) The exact contributions of D2S, D2L, D3, and D4 subreceptors could not be disentangled in the present study. (6) Without including molecular genetic indicators of functional dopaminergic properties (e.g., polymorphisms related to densities of pre-to postsynaptic D2 receptors; Zhang et al., 2007), it remains a data-based, plausible hypothesis to assume differential pre- to postsynaptic differences in high vs. low aE. Further pharamcogenetic studies including different substances (e.g., selective D2/D3 agonists/antagnonists, D4 antagonists) may help to elucidate and refine the model proposed here.

### Conclusions

Using resting posterior vs. anterior theta activity, we demonstrated that sulpiride's effects play out differently for individuals high and low in aE. Whereas the present findings cannot fully rule out that these differences are exclusively due to shifts in the time course of the drug responses, a more parsimonious model holds that low aE individuals are more sensitive to presynaptic, and high aE to postsynaptic sulpiride effects. These data not only add to the still limited evidence for a dopaminergic basis of aE, but also help to generate new hypotheses on the neurobiological mechanisms underlying the frequently observed paradoxical effects of dopaminergic drugs: pre- and postsynaptic reactivity depends on personality-correlated baseline dopamine levels. This factor contributes to the variability in the EEG-effects and possibly to the clinical efficacy of dopaminergic drugs. Future research may probe these suggestions and investigate the molecular basis of individual differences in pre- vs. postsynaptic dopamine D2 subreceptor densities and sensitivities.

### Conflict of interest statement

The authors declare that the research was conducted in the absence of any commercial or financial relationships that could be construed as a potential conflict of interest.

## Footnotes

^1^Although impulsivity is often viewed as a potential trait manifestation of dopamine (for recent research supporting this view see e.g., Dalley et al., 2007; Buckholtz et al., 2010) and as the trait resulting from individual differences in reward and incentive salience processing, empirically it seems to be more strongly related to serotonin (see reviews and data by Carver, 2005; Crockett et al., 2009; Robbins and Crockett, 2010). Moreover, it should be noted that the most common measures of impulsivity are heterogenous encompassing items assessing agentic extraversion and constraint (lower order traits like risk taking, novelty seeking, boldness, adventuresomeness, boredom susceptibility) or assessing motor and cognitive impulsivity. For this reason, measures of impulsivity are neither consistently highly interrelated, nor consistent in their correlation with extraversion (Depue and Collins, 1999). Thus, the Depue model only predicts significant empirical relations between impulsivity and dopamine for those measures of impulsivity more closely related to aE.

^2^In the past 20 years numerous studies aligned the dopamine receptor D4 gene and its polymorphisms to personality, althought there is only limited empirical evidence to assume a DRD4—aE or approach-related personality trait relation (for a recent meta-analysis see Munafo et al., 2008).
